# Serum anti-p53 antibodies in gastric adenocarcinoma patients are associated with poor prognosis, lymph node metastasis and poorly differentiated nuclear grade

**DOI:** 10.1038/sj.bjc.6690382

**Published:** 1999-05-01

**Authors:** C-W Wu, Y-Y Lin, G-D Chen, C-W Chi, D P Carbone, J-Y Chen

**Affiliations:** Institute of Biomedical Sciences, Academia Sinica, No. 128, Section 2, Yen-Chiu-Yuan Road, Taipei 11529, Taiwan, Republic of China; Departments of Surgery and Medical Research, Veterans General Hospital-Taipei; National Yang-Ming University, Taiwan, Republic of China; Division of Medical Oncology, Vanderbilt Cancer Center, University of Vanderbilt, Nashville, TN 37232-6838, USA

**Keywords:** gastric adenocarcinoma, serum anti-p53 antibodies, lymph node metastasis, nuclear grade, poor prognosis

## Abstract

Mutation of the *p53* tumour suppressor gene often leads to the accumulation of mutant p53 protein in tumour cells. Many cancer patients develop antibodies that recognize the overexpressed p53 protein. The presence of these antibodies is, in some tumour types, associated with poor prognosis. Gastric cancer is a highly prevalent disease associated with a high rate of mortality, there is a need for improved clinical and biological markers for disease behaviour. To investigate the clinical relevance of serum anti-p53 antibodies in patients with gastric adenocarcinoma, we have examined the sera of 501 gastric cancer patients for the presence of serum antibodies against the p53 protein. By immunoblotting analysis using a cell lysate containing overexpressed p53 protein as well as affinity-purified recombinant p53 protein as antigens, we have detected anti-p53 antibodies in 11.2% (61 of 501) of gastric cancer patients, but in none of 46 cancer-free individuals. The presence of anti-p53 antibodies was tightly associated with tumours of higher nuclear grade and lymph node metastasis, and a negative association was found between the presence of anti-p53 antibodies and survival. These results suggest that a preoperative test of serum anti-p53 antibodies in gastric cancer patients can be useful to identify subset of patients who may need gastrectomy with lymph node dissection and post-operative adjuvant therapy. © 1999 Cancer Research Campaign


*British Journal of Cancer* (1999) **80**(3/4), 483–488
© 1999 Cancer Research Campaign
Article no. bjoc.1998.0382

### Serum anti-p53 antibodies in gastric adenocarcinoma patients are associated with poor prognosis, lymph node metastasis and poorly differentiated nuclear grade

**C-W Wu[2,4], Y-Y Lin[1], G-D Chen[1], C-W Chi[3,4], DP Carbone[5] and J-Y Chen[1,4]**

[1]Institute of Biomedical Sciences, Academia Sinica, No. 128, Section 2, Yen-Chiu-Yuan Road, Taipei 11529, Taiwan, Republic of China; Departments of [2]Surgery and [3]Medical Research, Veterans General Hospital-Taipei; [4]National Yang-Ming University; Taiwan, Republic of China; [5]Division of Medical Oncology, Vanderbilt Cancer Center, University of Vanderbilt, Nashville, TN 37232-6838, USA

**Summary** Mutation of the *p53* tumour suppressor gene often leads to the accumulation of mutant p53 protein in tumour cells. Many cancer patients develop antibodies that recognize the overexpressed p53 protein. The presence of these antibodies is, in some tumour types, associated with poor prognosis. Gastric cancer is a highly prevalent disease associated with a high rate of mortality, there is a need for improved clinical and biological markers for disease behaviour. To investigate the clinical relevance of serum anti-p53 antibodies in patients with gastric adenocarcinoma, we have examined the sera of 501 gastric cancer patients for the presence of serum antibodies against the p53 protein. By immunoblotting analysis using a cell lysate containing overexpressed p53 protein as well as affinity-purified recombinant p53 protein as antigens, we have detected anti-p53 antibodies in 11.2% (61 of 501) of gastric cancer patients, but in none of 46 cancer-free individuals. The presence of anti-p53 antibodies was tightly associated with tumours of higher nuclear grade and lymph node metastasis, and a negative association was found between the presence of anti-p53 antibodies and survival. These results suggest that a preoperative test of serum anti-p53 antibodies in gastric cancer patients can be useful to identify subset of patients who may need gastrectomy with lymph node dissection and post-operative adjuvant therapy.

**Keywords**: gastric adenocarcinoma; serum anti-p53 antibodies; lymph node metastasis; nuclear grade; poor prognosis

Alterations of tumour suppressor gene *p53* is the most commonly observed genetic lesion in human cancers (Levine et al, 1991). *p53* alterations have a profound impact on tumour biology and the outcome of patients with many different tumour types. Most *p53* gene alterations are mis-sense mutations, and most of those lead to the synthesis of a mutant protein with a longer than normal half-life (Lane and Benchimol, 1990; Davidoff et al, 1991), and thus massive overexpression of the protein product. In many cases, this accumulated mutant p53 protein can induce a specific humoral response in patients with various types of cancer (Crawford et al, 1982; Caron de Fromentel et al, 1987; Winter et al, 1992; Lubin et al, 1993, 1995; Angelopoulou et al, 1994).

In general, anti-p53 antibodies have been found in cancer patients whose tumours contained p53 mis-sense mutations, but not in cancer-free populations, or in patients whose tumour had p53 splice/stop, splice, or frameshift mutations (Winter et al, 1992; Wild et al, 1995). These antibodies recognize both the wild-type and mutant p53 conformational and denaturation-resistant epitopes (Davidoff et al, 1992; Schlichtholz et al, 1992). Furthermore, naturally arising anti-p53 antibodies have been shown to recognize immunodominant epitopes in the carboxyl- and amino-terminal domains of the p53 polypeptide and these locations do not correspond to mutational hot-spots (Lubin et al, 1993; Wild et al, 1995). Although p53 immune responses have been observed in various types of tumours, it is important to note that only a proportion of the tumours that bear p53 mis-sense mutations induce antibodies (Angelopoulou et al, 1994). Thus other biological properties of the tumour and/or the host may allow the production of these responses and these other properties may have clinical significance.

*Received 28 May 1998*
*Revised 6 October 1998*
*Accepted 21 October 1998*

*Correspondence to:* J-Y Chen, Institute of Biomedical Sciences, Academia Sinica No. 128, Section 2, Yen-Chiu-Yuan Road, Taipei 11529, Taiwan, R.O.C..

The clinical implications of the development of serum anti-p53 antibodies in cancer patients has been controversial. Most studies show that the presence of such antibodies predicted a poor outcome. In breast cancer, patients who developed anti-p53 antibodies exhibited a shortened overall survival than those without anti-p53 antibodies, and the presence of anti-p53 antibodies was an independent prognostic factor (Schlichtholtz et al, 1992; Peyrat et al, 1995). In head and neck squamous cell carcinomas, the presence of anti-p53 antibodies was significantly associated with increased risks of relapse and death (Bourhis et al, 1996). On the other hand, in the newly diagnosed small-cell lung cancer patients the presence of anti-p53 antibodies was not associated with any clinical characteristics or prognostic markers (Rosenfeld et al, 1997). The clinical and biological relevance of anti-p53 antibodies in gastric cancer have not been previously studied in detail.

Gastric cancer is highly prevalent. It was estimated in 1990 to be the second most frequent cancer in the world (after lung cancer), with about 900 000 new cases diagnosed every year. In spite of a decreasing incidence, the prognosis for patients diagnosed with gastric cancer is dismal (Wu et al, 1997). Gastric cancer currently ranks as the second most common cause of cancer death and in some countries, especially those of the Far East such as China and Japan, it is the leading cause of cancer death. These factors have prompted the search for novel early diagnostic markers, improved therapies or prognostic factors that would enable a more effective treatment regimen to be tailored to specific groups of patients.

In gastric cancer, the *p53* gene is frequently mutated and *p53* overexpression is observed in about 50% of tumours (Kakeji et al, 1993; Motojima et al, 1994; Gabbert et al, 1995), and anti-p53 antisera were detected in some of the gastric cancer patients (Wurl et al, 1997). In this study, we examined a large number of gastric cancer patients for the presence of humoral antibodies against the tumour suppressor protein p53 and evaluated the clinical and biological significance of these antibodies in this disease by correlating their presence with the observed features of the tumour and clinical outcome.

### MATERIALS AND METHODS

### Patients and sera

Between February 1988 and August 1996, patients diagnosed with gastric adenocarcinoma who underwent gastric resection at the Veterans General Hospital (VGH)-Taipei were enrolled in this study. Patients with two primary tumours were excluded. A total of 501 patients were included based on fulfillment of the following criteria: (a) histologically confirmed disease, (b) available of preoperative serum, and (c) regular post-surgical follow-up. Control blood was obtained from forty-six cancer-free individuals during their physical check-up in the VGH-Taipei. Informed consent was obtained from each patient. Sera were prepared and stored at –80°C until use.

### p53 Antigens

A pCMV vector expressing human p53 mutant protein with a single amino acid change at codon 143 from valine to alanine was kindly provided by Dr Bert Vogelstein (Kern et al, 1992). The pCMV-p53V143A expression vector was introduced into human p53-null non-small-cell lung cancer (NSCLC) H1299 cells by electroporation (Chen et al, 1993). H1299 has a homozygous 5′-intragenic deletion of *p53* gene and produces no p53 mRNA or protein (Unger et al, 1992; Chen et al, 1993). Forty-eight hours post-transfection, cells were harvested. Cell lysate was prepared and used as the source of p53 antigen for immunoblot analysis.

Alternatively, bacterially expressed GST-p53 fusion protein was prepared for immunoblot analysis. cDNA encoding the full length p53 protein was in-frame fused to the bacterial glutathione S-transferase (GST) gene. GST and GST-p53 fusion proteins were expressed in *Escherichia coli* DH5α cells and affinity-purified as described (Sang et al, 1994).

### Immunoblot analysis

H1299 cell lysate containing overexpressed mutant p53 protein (V143A) and the affinity-purified GST-p53 fusion protein were used as p53 antigens for immunoblot analysis to detect the presence of anti-p53 antibodies in patients' sera (Chen et al, 1993). H1299 whole cell lysates or purified GST-p53 fusion protein were separated on 10% sodium dodecyl sulphate polyacrylamide gel electrophoresis (SDS-PAGE) using a mini-gel apparatus (Hoefer Mighty Small II). Proteins were then transferred to nitrocellulose filters (Schleicher and Schuell) followed by blocking with 5% non-fat dry milk. After drying, the nitrocellulose filters were cut into 3-mm strips and each strip was subjected to immunoblotting with each patient's serum (at 1:200 dilution) as primary and sheep anti-human Ig horseradish peroxidase (HRP) conjugates (at 1:500 dilution, Amersham, Inc.) as secondary antibodies. A colorimetric assay was employed to detect the presence of HRP by incubating the filter strips with phosphate-buffered saline (PBS) containing 3,3′-diaminobenzidine (0.5 mg $ml^{-1}$) and 0.015% hydrogen peroxide. Mouse anti-p53 monoclonal antibody α1801 (Ab-2, Oncogene Science) was included positive control (at final concentration of 1 μg $ml^{-1}$).

### Statistical analysis

The $\chi^2$ test and Student's *t*-test were adopted to evaluate clinicopathologic features that may relate to the status of p53 antibody development and the biological behaviour of the tumour. The following factors were considered: age, sex, tumour size, location of tumour, gross appearance (superficial, localized or infiltrative), nuclear grade, mode and depth of cancer invasion, Lauren's, Ming's and World Health Organization (WHO) histological classifications, stromal reaction pattern, lymphatic duct invasion, vascular invasion, lymph node metastasis, liver metastasis and peritoneal dissemination. To further analyse interactions between all possible factors, a backward step-wise logistic regression analysis was performed with anti-p53 antibodies as a dependent variable. We considered a $P$-value > 0.05 for likeness of fit in the final regression model. Survival curves were plotted using the Kaplan–Meier method. Comparisons were made by the generalized Wilcoxon and Mantel Cox tests. Multivariate survival analysis (BMDP P2L) was based on the Cox multiple step-wise regression model. A $P$-value < 0.05 was considered to be statistically significant. Computation was carried out using BMDP statistical software (Dixon, 1988).

### RESULTS

### Detection of serum anti-p53 antibodies in gastric cancer patients

We evaluated the sera from 501 patients with gastric cancer for the presence of anti-p53 antibodies. Immunoblot analysis was performed using the patient sera as primary antibodies against cell lysates prepared from a p53-null human lung cancer H1299 cell line transfected with a pCMV vector expressing mutant p53 (with amino acid change at codon 143 from valine to alanine) and the same line transfected with the parental plasmid containing no p53 cDNA insert (Kern et al, 1992; Chen et al, 1993). After transfection with pCMV-p53V143A, H1299 cells expressed a substantial amount of p53 protein which was readily detected by immunoblot analyses with mouse anti-p53 monoclonal antibody α1801 (Figure 1). Sera from some of the gastric cancer patients also detected a band with a migration distance similar to that of p53 protein. The sera scored as positive were those detecting a 53 kDa signal when blotted against the lysate prepared from pCMV-p53V143A transfectants but no signal from identically prepared mock-transfected

 © Cancer Research Campaign 1999

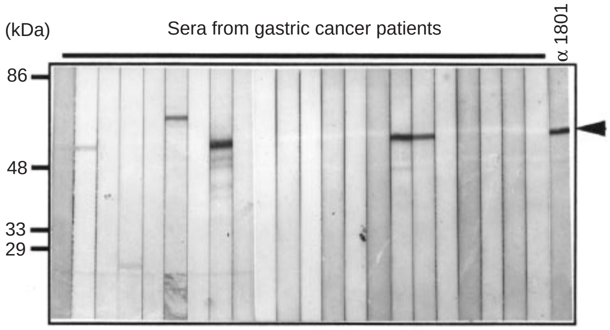

**Figure 1** Gastric cancer patients generate humoral antibodies against tumour suppressor p53. Protein extracts (100 µg per preparative gel) prepared from NSCLC-H1299 cells expressing a mutant p53 protein were analysed on a 10% SDS-PAGE gel for immunoblot analysis. After transblotting, the nitrocellulose filter was cut into strips and each strip was blotted against a single patient's serum (at a 1:200 dilution). Sheep anti-human and sheep anti-mouse Ig HRP conjugates were used as secondary antibodies respectively. The presence of HRP was detected colorimetrically. The far right lane represents a positive control (α1801) and p53 signal was indicated by an arrowhead. Signals with a molecular weight other than 53 kDa were also detected in some patients' sera on a less frequent basis

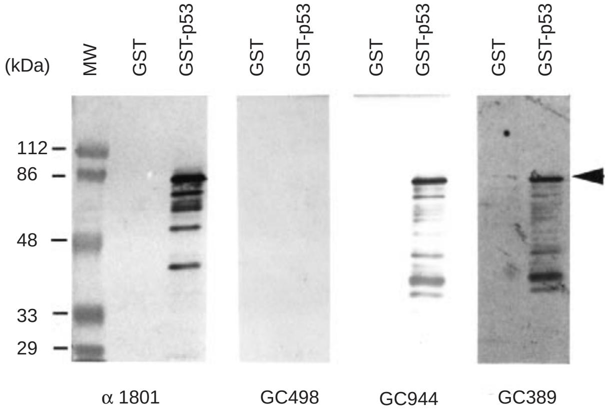

**Figure 2** Gastric cancer patients developed circulating antibodies specific for tumour suppressor p53. Affinity-purified GST and GST-p53 fusion proteins (5 µg protein per lane) were separated by 10% SDS-PAGE and immunodetected with sera from different patients (GC944, GC389 and GC498) (at 1:500 dilution) as well as mouse anti-p53 monoclonal antibody α 1801. HRP-conjugated sheep anti-human or mouse Ig were used as secondary antibodies (at 1:5000 dilution). HRP was detected by enhanced chemoluminescence (Amersham, Inc.). The signal of GST-p53 fusion protein (86 kDa) was indicated by the arrowhead cells or cells transfected with the parental pCMV vector. To independently confirm that the immune response observed was directed against the tumour suppressor protein p53, immunoblot analysis was carried out using affinity-purified GST-p53 protein as antigen. All of the sera which detected the 53 kDa signal against the H1299 transfectant were shown to recognize the 80 kDa GST-p53 fusion protein but not GST protein itself, suggesting that the immune response was specific for the tumour suppressor p53 protein. Figure 2 shows the immunoblot analysis of GST and GST-p53 fusion proteins with two p53 antibody-positive (from patients GC944 and GC389) and one antibody-negative (GC498) sera. Sera from 61 (12.2%) of the 501 gastric cancer patients yielded a positive signal toward tumour suppressor p53 protein by this assay. In contrast, none of the control sera obtained from 46 cancer-free individuals were shown to contain anti-p53 antibodies, consistent with the extremely low incidence of anti-p53 antibodies in normal individuals in other reports.

These anti-p53 antibodies elicited in gastric cancer patients were able to recognize and form complexes with p53 proteins of both the wild-type and mutant conformations as evidenced by immunoprecipitation of [$^{35}$S]-methionine-labelled p53 proteins synthesized in vitro using a rabbit reticulocyte lysate system by p53-antibody-positive sera (data not shown).

### Correlation between anti-p53 antibodies and clinicopathologic status

The correlation of anti-p53 antibodies to clinical and tumour biological parameters was then analysed. As shown in Table 1, univariate analysis demonstrated that the presence of anti-p53 antibodies is significantly associated with several factors including large tumour size, ill-defined gross appearance, poorly differentiated nuclear grade, increased depth of invasion, lymphatic duct invasion and lymph node metastasis. No correlation was found between the presence of anti-p53 antibodies and the other parameters tested. Logistic regression model ($P = 1.000$ for goodness of fit) revealed that lymph node metastasis ($P = 0.002$) and nuclear grade ($P = 0.003$) were the factors independently associated with the development of these antibodies (data not shown).

All the 501 patients included in this study received only surgical resection without adjuvant or other types of therapy. The cumulative 5-year survival was 51% in this series. As shown in Figure 3, there was a significantly poorer survival rate for patients with anti-p53 antibodies compared to those without them ($P = 0.0038$). The median survival of p53-antibody-negative patients was more than 60 months while it was 24 months for p53-antibody-positive patients. This was also reflected in the 5-year survival rates of 53% and 31% respectively. Table 2 shows the 5-year survival rates according to each parameter in the 501 patients. Anti-p53 antibodies ($P < 0.0038$), along with other tumour- and host-related factors are associated with decreased survival. These factors include tumour size ($P < 0.0001$), ill-defined gross appearance ($P < 0.0001$), poorly differentiated nuclear grade ($P = 0.0002$), expansive type of invasion and invasion to or through the serosa ($P \leq 0.0001$), higher grade in the three commonly used histopathologic classifications ($P \leq 0.01$), stromal reaction ($P < 0.0001$), lymphatic and haematogenous spread ($P \leq 0.0001$), lymph node metastasis ($P < 0.0001$), liver metastasis ($P < 0.0001$) and peritoneal dissemination ($P < 0.0001$). To identify independent factors that would predict patients' outcome, a multivariate analysis was performed using the Cox regression model. Depth of tumour invasion ($P < 0.0001$), poorly differentiated histology (by WHO classification) ($P = 0.03$), venous invasion ($P = 0.0009$), lymph node metastasis ($P < 0.0001$), liver metastasis ($P < 0.0001$) and peritoneal dissemination ($P = 0.0099$) emerged as independent prognostic parameters.

### DISCUSSION

Development of tumour-associated antibodies against dominant and recessive oncogenes has been observed in several other tumour types (Caron de Fromentel et al, 1987; Ben-Mahrez et al, 1990; Sorokine et al, 1991; Winter et al, 1992; Angelopoulou et al, 1994), and in some cases, the presence of anti-p53 antibodies is

© Cancer Research Campaign 1999 

**Table 1** Correlation between the status of serum anti-p53 antibodies to clinicopathological features of gastric cancer patients

| | Negative *n* = 440 (87.8%) | Positive *n* = 61 (12.2%) | *P*-value |
|---|---|---|---|
| Age (year) | 66.3 ± 10.5 | 67.5 ± 9.3 | 0.3390 |
| Sex | | | |
| Men | 356 | 55 | |
| Women | 84 | 6 | 0.0776 |
| Tumour size (cm, mean ± s.d) | 5.7 ± 3.4 | 7.5 ± 2.9 | < 0.0001[c] |
| Location of tumour | | | |
| Upper (C) | 64 | 6 | |
| Middle (M) | 143 | 21 | |
| Lower (A) | 233 | 34 | 0.6089 |
| Gross appearance | | | |
| Superficial | 139 | 7 | |
| Localized | 62 | 12 | |
| Infiltrative | 239 | 42 | 0.0052[c] |
| Nuclear grade[a] | | | |
| Well-differentiated | 19 | 0 | |
| Moderately differentiated | 171 | 12 | |
| Poorly differentiated | 250 | 49 | 0.0015[c] |
| Mode of invasion | | | |
| Expansive | 229 | 32 | |
| Intermediate | 122 | 20 | |
| Infiltrative | 89 | 9 | 0.5205 |
| Depth of invasion[b] | | | |
| m, sm | 124 | 7 | |
| pm, ss | 73 | 10 | |
| se, si | 243 | 44 | 0.0151[c] |
| Japanese histologic type | | | |
| Papillary + Tubular | 201 | 32 | |
| Poorly + Signet + Mucinous | 239 | 29 | 0.3199 |
| Lauren's classification | | | |
| Intestinal type | 230 | 22 | |
| Diffuse type | 191 | 35 | |
| Unclassified | 19 | 4 | 0.0579 |
| Ming's classification | | | |
| Expanding type | 152 | 22 | |
| Infiltrative type | 288 | 39 | 0.8152 |
| Stromal reaction pattern | | | |
| Medullary | 154 | 23 | |
| Intermediate | 159 | 21 | |
| Schirrhous | 127 | 17 | 0.9171 |
| Lymphatic duct invasion | | | |
| Negative | 129 | 7 | |
| Positive | 311 | 54 | 0.0033[c] |
| Venous invasion | | | |
| Negative | 414 | 55 | |
| Positive | 26 | 6 | 0.2594 |
| Lymph node metastasis | | | |
| Negative | 184 | 10 | |
| Positive | 256 | 51 | 0.0001[c] |
| Liver metastasis | | | |
| Negative | 418 | 56 | |
| Positive | 22 | 5 | 0.3568 |
| Peritoneal dissemination | | | |
| Negative | 397 | 52 | |
| Positive | 43 | 9 | 0.2319 |

[a]Nuclear grade was determined according to nuclear pleomorphism of tumour cells, well differentiated: nuclei with minimal variation in size and shape; moderately differentiated: nuclei with moderate variation in size and shape; poorly differentiated: nuclei with marked variation in size and shape
[b]Depth of invasion. m: mucosa; sm: submucosa; pm: proper muscle; ss: subserosa; se: serosa exposed; si: serosa infiltrated .[c]Statistically significant.

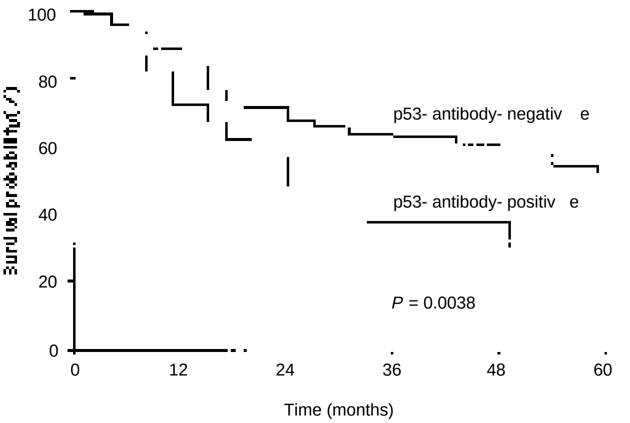

**Figure 3** Survival curves for gastric cancer patients with and without serum anti-p53 antibodies correlated to poor prognosis (Schlichtholz et al, 1992; Peyrat et al, 1995; Bourhis et al, 1996). The present study is the first to study a large number of patients with primary gastric adenocarcinoma and determine the frequency at which they generate circulating antibodies against p53, and to correlate the development of antibodies with relevant clinical and biological parameters. Sixty-one of 501 (12.2%) patients were shown to develop these antibodies at levels sufficient to be detected by the Western blot assay used here. The presence of serum anti-p53 antibodies in patients with gastric adenocarcinoma was associated with tumour- and host-related factors including large tumour size, ill-defined tumour margins, poorly differentiated nuclear grade, depth of invasion, lymphatic duct invasion and lymph node metastasis. Anti-p53 antibodies were associated with a poor patient survival. A logistic regression model revealed that lymph node metastasis ($P$ = 0.002) and nuclear grade ($P$ = 0.003) were the factors independently associated with the development of these antibodies.

It has been reported that mutation of the *p53* gene occurs in approximately 50% of gastric tumours (Kakeji et al, 1993; Motojima et al, 1994). Our data suggest that approximately 20–30% of gastric cancer patients with tumours bearing *p53* gene mutations develop anti-p53 antibodies. This is consistent with previous observations in patients with other types of cancers that approximately this proportion of patients with mutations develop anti-p53 antibodies (Angelopoulou et al, 1994). Thus, not all of the tumours bearing p53 mis-sense mutations induce anti-p53 antibodies, suggesting factors other than simple overexpression of the p53 protein are involved in eliciting the immune response (Angelopoulou et al, 1994). The fact that 84% (51 of 61) of the p53-antibody-positive patients displayed lymph node metastasis suggests that involvement of lymph nodes has hitherto unrecognized importance in eliciting the immune response against overexpressed p53 protein, and that immunological mechanisms by which the development of anti-p53 antibodies may signify an aggressive subset of tumours with p53 mutations with the propensity to involve lymph nodes.

Gastric carcinoma is a world-wide disease with a dismal prognosis. Although surgical resection offers the only prospect of cure, long-term survival of patients with adenocarcinoma of the stomach remains poor because of invasion of the tumour through the muscular layer (advanced cancer) and early lymphatic spread,

 © Cancer Research Campaign 1999

**Table 2** Association between 5-year survival rates and clinicopathological features of gastric cancer patients

| Parameter | No. of patients (total *n* = 501) | 5-year survival (%) | Univariate *P*-value | Multivariate *P*-value |
|---|---|---|---|---|
| Age | | | | |
| ≤ 65 | 188 | 54.8 | | |
| ≥ 65 | 313 | 48.6 | 0.0952 | 0.5457 |
| Sex | | | | |
| Men | 411 | 48.5 | | |
| Women | 90 | 62.7 | 0.1252 | 0.9472 |
| Tumour size | | | | |
| < 4 cm | 162 | 83.1 | | |
| 4–8 cm | 235 | 41.1 | | |
| > 8 cm | 104 | 26.6 | < 0.0001[c] | 0.2951 |
| Location of tumour | | | | |
| Upper (C) | 70 | 59.9 | | |
| Middle (M) | 164 | 53.3 | | |
| Lower (A) | 267 | 47.2 | 0.3454 | 0.8470 |
| Gross appearance | | | | |
| Superficial | 146 | 90.3 | | |
| Localized | 74 | 64.1 | | |
| Infiltrative | 281 | 27.9 | < 0.0001[c] | 0.0748 |
| Nuclear grade[a] | | | | |
| Well-differentiated | 19 | 62.2 | | |
| Moderately differentiated 183 | 65.4 | | | |
| Poorly differentiated | 299 | 42.0 | 0.0002[c] | 0.1382 |
| Mode of invasion | | | | |
| Expansive | 261 | 38.1 | | |
| Intermediate | 142 | 66.3 | | |
| Infiltrative | 98 | 67.6 | 0.0001[c] | 0.4905 |
| Depth of invasion[b] | | | | |
| m, sm | 131 | 95.3 | | |
| pm, ss | 83 | 72.0 | | |
| se, si | 287 | 25.0 | < 0.0001[c] | < 0.0001[c] |
| Japanese histologic type | | | | |
| Papillary + Tubular | 233 | 62.3 | | |
| Poorly + Signet + Mucinous | 268 | 35.2 | 0.0001[c] | 0.0320[c] |
| Lauren’s classification | | | | |
| Intestinal type | 252 | 63.5 | | |
| Diffuse type | 226 | 38.1 | | |
| Unclassified | 23 | 47.1 | 0.0002[c] | 0.3811 |
| Ming’s classification | | | | |
| Expanding type | 174 | 65.5 | | |
| Infiltrative type | 327 | 43.6 | 0.0110[c] | 0.1723 |
| Stromal reaction pattern | | | | |
| Medullary | 177 | 55.1 | | |
| Intermediate | 180 | 25.4 | | |
| Schirrhous | 144 | 72.0 | < 0.0001[c] | 0.1443 |
| Lymphatic duct invasion | | | | |
| Negative | 136 | 85.4 | | |
| Positive | 365 | 37.7 | < 0.0001[c] | 0.7611 |
| Venous invasion | | | | |
| Negative | 469 | 53.0 | | |
| Positive | 32 | 23.0 | 0.0001[c] | 0.0009[c] |
| Lymph node metastasis | | | | |
| Negative | 194 | 87.6 | | |
| Positive | 307 | 29.1 | < 0.0001[c] | < 0.0001[c] |
| Liver metastasis | | | | |
| Negative | 474 | 53.3 | | |
| Positive | 27 | 0.0 | < 0.0001[c] | < 0.0001[c] |
| Peritoneal dissemination | | | | |
| Negative | 449 | 56.6 | | |
| Positive | 52 | 0.0 | < 0.0001[c] | 0.0099[c] |
| p53 antibodies | | | | |
| Negative | 440 | 53.2 | | |
| Positive | 61 | 31.3 | 0.0038* | 0.6396 |

[a]Nuclear grade was determined according to nuclear pleomorphism of tumour cells, well differentiated: nuclei with minimal variation in size and shape; moderately differentiated: nuclei with moderate variation in size and shape; poorly differentiated: nuclei with marked variation in size and shape. [b]Depth of invasion. m: mucosa; sm: submucosa; pm: proper muscle; ss: subserosa; se: serosa exposed; si: serosa infiltrated. [c]Statistically significant.

*© Cancer Research Campaign 1999*

possibly resulting in residual microscopic disease and relapse after surgical resection (Wu et al, 1996*b*, 1996*c*). In this study, 74% of the tumours were advanced carcinomas and 61% of the patients had lymph node metastases. Since regional lymph node and recurrent nodal involvement have an adverse effect on survival (Wu et al, 1996*a*), gastric resection as well as removal of metastatic lymph nodes are generally considered when attempting curative resection of adenocarcinomas of the stomach. However, the possibility of increased operative risk through the extension of lymphadenectomy with controversial clinical benefit raises concerns about this procedure. In this study, lymph node metastasis and poorly differentiated nuclear grade are independently associated with these antibodies in multivariate analysis. Kakeji et al have reported that gastric cancer with p53 overexpression is highly proliferative and has a high potential for metastasizing to lymph nodes (Kakeji et al, 1993). If lymph node involvement precedes the development of anti-p53 antibodies, detection of antibodies would not be a useful early cancer marker as has been suggested for lung cancer patients (Lubin et al, 1995). The fact that only 12.2% of gastric cancer patients were sero-positive for p53-antibodies further excludes its global use as an early diagnostic marker for this disease. On the other hand, the identification of patients with perhaps occult lymph node metastasis and thus poor prognosis prior to surgical and histopathological detection of metastasis is in itself a worthwhile goal. In contrast to other preoperative image diagnostics, including endoscopic sonography and computerized tomography, the serological assay of anti-p53 antibodies is non-invasive and easy to perform, and may help to pinpoint the subset of patients who should be subjected to gastrectomy with lymph node dissection, or it could also be used along with other established parameters to identify subsets of patients with poor prognosis who may need post-operative adjuvant therapy.

### ACKNOWLEDGEMENTS

This work was supported by grants in part from the National Science Council (NSC84-2331-B-001-035) and the Department of Health, Taiwan, Republic of China. We thank Dr Duncan Smith for his comments on the manuscript.

### REFERENCES

Angelopoulou K, Diamandis EP, Sutherland DJA, Kellen JA and Bunting PS (1994) Prevalence of serum antibodies against the *p53* tumor suppressor gene protein in various cancers. *Int J Cancer* **58**: 480–487

Ben-Mahrez K, Sorokine I, Thierry D, Kawasumi T, Ishii S, Salmon R and Kohiyama M (1990) Circulating antibodies against c-*myc* oncogene product in sera of colorectal cancer patients. *Int J Cancer* **46**: 35–38

Bourhis J, Lubin R, Roche B, Koscielny S, Bosq J, Dubois I, Talbot M, Marandas P, Schwaab G, Wibault P, Luboinski B, Eschwege F and Soussi T (1996) Analysis of p53 serum antibodies in patients with head and neck squamous cell carcinoma. *J Natl Cancer Inst* **88**: 1228–1233

Caron de Fromentel C, May-Levine F, Mouriesse H, Lemerle J, Chandresekaran K and May P (1987) Presence of circulating antibodies against the cellular protein p53 in a notable proportion of children with B cell lymphoma. *Int J Cancer* **39**: 185–189

Chen J-Y, Funk WD, Wright WE, Shay JW and Minna JD (1993) Heterogeneity of transcriptional activity of mutant p53 proteins and p53 DNA target sequences. *Oncogene* **8**: 2159–2166

Crawford L, Pim D and Bulbrook R (1982) Detection of antibodies against the cellular protein p53 in sera from patient with breast cancer. *Int J Cancer* **30**: 403–408

Davidoff A, Humphrey P, Iglehart J and Marks J (1991) Genetic basis for p53 overexpression in human breast cancer. *Proc Natl Acad Sci USA* **88**: 5006–5010

Davidoff AM, Iglehart JD and Marks JR (1992) Immune response to p53 is dependent upon p53/HSP70 complexes in breast cancers. *Proc Natl Acad Sci USA* **89**: 3439–3442

Dixon WJ (1988) *BMDP Statistical Software Manual*. University of California Press: Berkeley

Gabbert HE, Muller W, Schneiders A, Meier S and Hommel G (1995) The relationship of p53 expression to the prognosis of 418 patients with gastric carcinoma. *Cancer* **76**: 720–726

Kakeji Y, Korenaga D, Tsujitani S, Baba H, Anai H, Maehara Y and Sugimachi K (1993) Gastric cancer with p53 overexpression has high potential for metastasising to lymph nodes. *Br J Cancer* **67**: 589–593

Kern S, Pietenpol JA, Thiagalingam S, Seymour A, Kinzler KW and Vogelstein B (1992) Oncgenic forms of p53 inhibit p53-regulated gene expression. *Science* **256**: 827–830

Lane D and Benchimol S (1990) p53: oncogene or anti-oncogene? *Genes Dev* **4**: 1–8

Levine A, Momand J and Finlay C (1991) The p53 tumour suppressor gene. *Nature* **351**: 453–455

Lubin R, Schlichtholz B, Bengoufa D, Zalcman G, Tredaniel J, Hirsch A, Caron de Fromental C, Preudhomme C, Fenaux P, Fournier G, Mangin P, Laurent-Puig P, Pelletier G, Schlumberger M, Desgrandchamps F, Le Duc A, Peyrat JP, Janin N, Bressac B and Soussi T (1993) Analysis of p53 antibodies in patients with various cancers define B-cell epitopes of human p53: Distribution on primary structure and exposure on protein surface. *Cancer Res* **53**: 5872–5876

Lubin R, Zalcman G, Bouchet L, Tredaniel J, Legros Y, Cazals D, Hirsch A and Soussi T (1995) Serum p53 antibodies as early markers of lung cancer. *Nat Med* **1**: 701–702

Motojima K, Furui J, Kohara N, Ito T and Kanematsu T (1994) Expression of p53 protein in gastric carcinoma is not independently prognostic. *Surgery* **116**: 890–895

Peyrat JP, Bonneterre J, Lubin R, Vanlemmens L, Fournier J and Soussi T (1995) Prognostic significance of circulating p53 antibodies in patients undergoing surgery for locoregional breast cancer. *Lancet* **345**: 621–623

Rosenfeld MR, Malats N, Schramm L, Graus F, Cardenal F, Vinolas N, Rosell R, Tora M, Real FX, Posner JB and Dalmau J (1997) Aerum anti-p53 antibodies and prognosis of patients with small-cell lung cancer. *J Natl Cancer Inst* **89**: 381–385

Sang BC, Chen J-Y, Minna JD and Barbosa MS (1994) Distinct regions of p53 have a differential role in transcriptional activation and repression functions. *Oncogene* **9**: 853–859

Schlichtholz B, Legros Y, Gillet D, Gaillard C, Marty M, Lane D, Calvo F and Soussi T (1992) The immune response to p53 in breast cancer patients is directed against immunodominant epitopes unrelated to the mutational hot spot. *Cancer Res* **52**: 6380–6384

Sorokine I, Ben-Mahrez K, Bracone A, Thierry D, Ishii S, Imamoto F and Kohiyama M (1991) Presence of circulating anti-c-myb oncogene product antibodies in human sera. *Int J Cancer* **47**: 665–669

Unger T, Nau M, Segal S and Minna J (1992) p53: a transdominant regulator of transcription whose function is ablated by mutations occurring in human cancer. *EMBO J* **11**: 1383–1390

Wild CP, Ridanpaa M, Anttila S, Lubin R, Soussi T, Husgafvel-Pursiainen K and and Vainio H (1995) p53 antibodies in the sera of lung cancer patients: comparison with p53 mutation in the tumor tissue. *Int J Cancer* **64**: 176–181

Winter SF, Minna JD, Johnson BE, Takahashi T, Gardar AF and Carbone DP (1992) Development of antibodies against p53 in lung cancer patients appears to be dependent on the type of p53 mutation. *Cancer Res* **52**: 4168–4174

Wu C-W, Hsieh M-C, Lo S-S, Lui W-Y and P'eng F-K (1996*a*) Results of curative gastrectomy for carcinoma of the distal third of the stomach. *J Am Coll Surg* **183**: 201–207

Wu C-W, Hsieh M-C, Lo S-S, Tsay S-H, Lui W-Y and P'eng F-K (1996*b*) Relation of number of positive lymph nodes to the prognosis of patients with primary gastric adenocarcinoma. *Gut* **38**: 525–527

Wu C-W, Hsieh M-C, Lo S-S, Tsay S-H, Lui W-Y and P'eng F-K (1996*c*) Lymph node metastasis from carcinoma of the distal one-third of the stomach. *Cancer* **73**: 2059–2064

Wu C-W, Hsieh M-J, Lo S-S, Tsay S-H, Li AF-Y, Lui W-Y and P'eng F-K (1997) Prognostic indicators for survival after curative resection for patients with carcinoma of the stomach. *Dig Dis Sci* **42**: 1265–1269

Wurl P, Weigmann F, Meye A, Fiittkau M, Rose U, Berger D, Rath F-W, Dralle H and Taubert H (1997) Detection of p53 autoantibodies in sera of gastric cancer patients and their prognostic relevance. *Scand J Gastroentrol* **32**: 1147–1151

© Cancer Research Campaign 1999
